# The Central Role of Imaging in Renal Cell Carcinoma: A Comprehensive Review of Tumor Aggressiveness, Histology, and Radiomics

**DOI:** 10.3390/cancers18132129

**Published:** 2026-06-30

**Authors:** Andreu Ivars, Blanca Paño, Josep Puig, María Fresno, Leonardo Rodríguez, Carmen Sebastia, Carlos Nicolau

**Affiliations:** 1 Radiology Department, Clinic Hospital, IDIBAPS, University of Barcelona (UB), 08036 Barcelona, Spain; bpano@clinic.cat (B.P.); jpuig2@clinic.cat (J.P.); mfresno@clinic.cat (M.F.); msebasti@clinic.cat (C.S.); cnicolau@clinic.cat (C.N.); 2 Pathology Department, Clinic Hospital, IDIBAPS, University of Barcelona (UB), 08036 Barcelona, Spain; lerodrig@clinic.cat

**Keywords:** renal cell carcinoma, computed tomography, tumor aggressiveness, histologic subtypes, radiologic–pathologic correlation, ISUP grade, radiomics, quantitative imaging, renal mass

## Abstract

Renal cell carcinoma encompasses a heterogeneous group of kidney tumors with wide variations in biological behavior, histologic subtype, and clinical aggressiveness. Accurate preoperative characterization is essential for management, yet remains challenging because benign, indolent, and aggressive lesions often show overlapping imaging features. CT is the most widely used modality for renal mass evaluation, offering broad availability, high spatial resolution, and multiphasic acquisition. This comprehensive narrative review summarizes current evidence on CT-based characterization of renal cell carcinoma, radiologic–pathologic correlation, imaging markers of tumor aggressiveness, renal mass biopsy, and radiomics. Radiomics and other quantitative imaging approaches may complement conventional imaging by capturing tumor heterogeneity and biologically relevant features; however, most studies remain retrospective, single-center, and methodologically heterogeneous. Multicenter validation, standardized acquisition and segmentation, transparent reporting, and demonstration of added clinical value are required before these methods can be implemented in routine care.

## Simple Summary

Renal cell carcinoma is a common kidney cancer with a broad spectrum of behavior, ranging from indolent tumors to highly aggressive disease. Computed tomography (CT) is central to preoperative assessment because it is widely available and provides detailed information on tumor morphology, enhancement, local extension, and staging. However, imaging alone cannot always determine tumor subtype or aggressiveness. This comprehensive narrative review summarizes how CT and complementary imaging findings relate to renal cell carcinoma biology, histologic subtype, and tumor grade. It also discusses renal mass biopsy and emerging quantitative approaches, including radiomics and artificial intelligence, while emphasizing that these tools remain adjuncts requiring standardization and external validation before routine clinical adoption.

## 1. Literature Search Strategy and Review Scope

This article was conceived as a comprehensive narrative review rather than a systematic review or meta-analysis. Relevant literature was identified through targeted searches of PubMed, Scopus, and Web of Science using combinations of the terms “renal cell carcinoma”, “computed tomography”, “radiomics”, “tumor aggressiveness”, “histologic subtype”, “ISUP grade”, “renal mass biopsy”, “molecular imaging”, “photon-counting CT”, “artificial intelligence”, and “imaging biomarkers”. Priority was given to clinical guidelines, landmark radiology and pathology studies, recent original investigations, systematic reviews or meta-analyses when available, and high-quality narrative reviews directly relevant to CT-based characterization, radiologic–pathologic correlation, and quantitative imaging biomarkers in RCC.

Because the review has a narrative scope, no systematic-review protocol was registered, no PRISMA flow diagram was generated, and formal inclusion/exclusion criteria were not applied. The evidence is therefore summarized and contextualized for clinical interpretation rather than quantitatively synthesized.

## 2. Introduction

Renal cell carcinoma (RCC) accounted for nearly half a million new cases worldwide in 2022 and remains one of the most common urologic malignancies. Some cases are hereditary, but most are sporadic [1]. Its incidence has steadily increased, largely due to the widespread use of cross-sectional imaging, which has led to the incidental detection of many asymptomatic renal masses. Advanced-stage disease often exhibits clinical symptoms or paraneoplastic manifestations, although early diagnosis and therapeutic improvements have reduced mortality rates [2]. Globally, about 70% of cases are diagnosed at stage I, while 11% have metastatic disease (stage IV) [3].

However, many incidental tumors show aggressive behavior or unfavorable histology. RCC comprises a heterogeneous group of tumors with distinct histologic, molecular, and clinical characteristics affecting survival. Clear cell RCC (ccRCC) is the predominant subtype, comprising 75–80% of cases. The remaining 20–25% are non-clear cell RCCs, including papillary and chromophobe subtypes, and a few other entities. Chromophobe tumors have the most favorable prognosis, with 5-year survival rates exceeding 80–90% [4]. Papillary tumors show better outcomes than ccRCC, though outcomes are less favorable for type 2 versus type 1 papillary RCC [5]. Histologic subtype, tumor grade, necrosis, lymph node involvement, and sarcomatoid differentiation are among the most important prognostic factors, correlating with recurrence and survival. However, most variables are usually determined after surgery, limiting their value for preoperative risk stratification.

Imaging plays a central role in the evaluation of renal tumors. Current guidelines from the American College of Radiology and the American Urological Association emphasize the importance of imaging for lesion detection, characterization, staging, and treatment planning [6,7] (Table 1). Ultrasound is frequently used as an initial tool because of its availability and safety profile, while contrast-enhanced ultrasound (CEUS) can provide additional information regarding lesion vascularity and enhancement patterns. Nevertheless, ultrasound remains limited for comprehensive tumor characterization and staging. Multiphasic contrast-enhanced computed tomography (CT) is the standard imaging modality for RCC evaluation because of its widespread availability, high spatial resolution, and reproducibility. CT provides essential information regarding tumor morphology, local extension, vascular involvement, and metastatic disease, making it indispensable for staging and surgical planning [3,8,9]. Dual-energy CT (DECT) has further expanded the capabilities of conventional CT by enabling material decomposition and quantitative iodine assessment, although its role in biological risk stratification remains under investigation [10]. Multiparametric MRI offers superior functional information through dynamic contrast-enhanced (DCE) and diffusion-weighted imaging (DWI), to improve discrimination between benign and malignant renal lesions [11,12]. High-grade ccRCCs show lower apparent diffusion coefficient (ADC) values on DWI, indicating increased cellularity, and reduced wash-in and enhancement indices on DCE imaging compared to low-grade tumors. Quantitative parameters such as the tumor-to-cortex ADC ratio enhance differentiation, with lower ratios consistently associated with high-grade disease [13,14,15]. Current guidelines, including the American College of Radiology, recommend MRI as a complementary problem-solving tool for indeterminate renal masses or when CT findings are equivocal [8].

Despite major advances in imaging technology, reliable preoperative prediction of histologic subtype and tumor aggressiveness remains challenging. Considerable overlap exists among benign lesions, indolent tumors, and aggressive RCC subtypes. Consequently, there is increasing interest in quantitative imaging approaches capable of extracting biologically relevant information from routine imaging studies. In this review, we summarize the current role of CT in RCC characterization, discuss imaging features associated with tumor aggressiveness and histologic subtype, and explore the emerging contribution of radiomics to non-invasive tumor assessment [16,17].

## 3. Histologic Subtypes, Tumor Aggressiveness and ISUP Grading in Renal Cell Carcinoma

### 3.1. Histologic Subtypes and Pathologic Assessment of RCC

RCC encompasses a diverse group of epithelial malignancies with distinct biological behavior and clinical outcomes. The principal histologic patterns include clear cell, papillary, chromophobe, collecting duct, and unclassified morphologies. The three major histologic subtypes account for the vast majority of cases: clear cell RCC (75–80%), papillary RCC (10–15%), and chromophobe RCC (approximately 5%) [1,3].

Clear cell RCC is generally associated with more aggressive biological behavior and accounts for most metastatic cases. Papillary RCC typically demonstrates intermediate clinical behavior, whereas chromophobe RCC is often associated with a more favorable prognosis. Less common entities, including collecting duct carcinoma and unclassified RCC, tend to exhibit aggressive clinical features and are frequently diagnosed at advanced stages [18].

Histologic subtype remains an important determinant of prognosis and treatment strategy. However, definitive classification is usually established after tissue sampling or surgical resection, highlighting the need for reliable imaging biomarkers capable of improving preoperative tumor characterization.

### 3.2. Histopathologic Features of Tumor Aggressiveness

**Figure 1:**
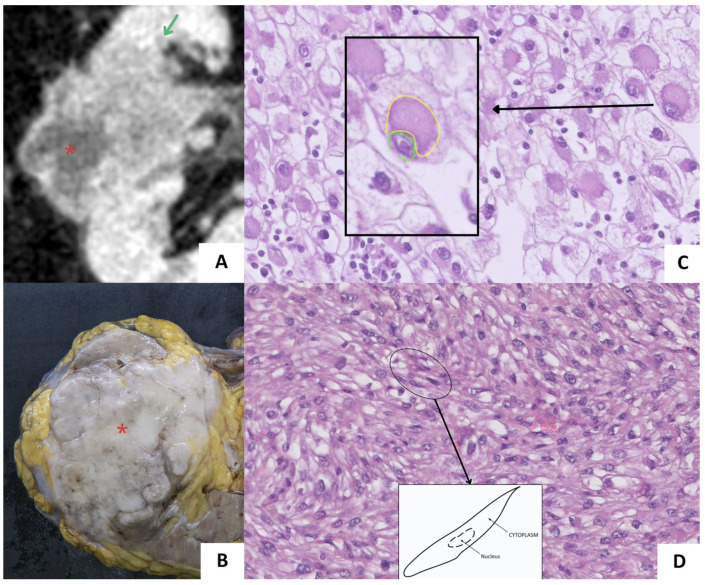
Imaging and histopathologic examples of tumor dedifferentiation. (**A**,**B**) RCC with central necrosis (*) on CT and gross pathology. (**C**) Rhabdoid differentiation. Black arrow connecting to a higher-magnification inset (black box), with an individual cell showing abundant cytoplasm (yellow outline) and prominent eccentric nucleus (green outline). (**D**) Sarcomatoid differentiation. Black oval outlines a cluster of spindle cells, with a black arrow leading to a schematic diagram of the typical sarcomatoid cell morphology.

In addition to histologic subtype, several pathologic features are closely associated with tumor aggressiveness in RCC. These include nuclear atypia, tumor necrosis, and sarcomatoid or rhabdoid differentiation (Figure 1). High-grade tumors typically exhibit prominent nucleoli, marked nuclear pleomorphism, and varying degrees of dedifferentiation.

Sarcomatoid differentiation may occur in any RCC subtype and is characterized by spindle-cell morphology resembling high-grade sarcoma. Rhabdoid differentiation is defined by large epithelioid cells with eccentric nuclei and abundant eosinophilic cytoplasm. Both features are associated with aggressive clinical behavior, early metastatic spread, and poor oncologic outcomes [19,20].

**Figure 2:**
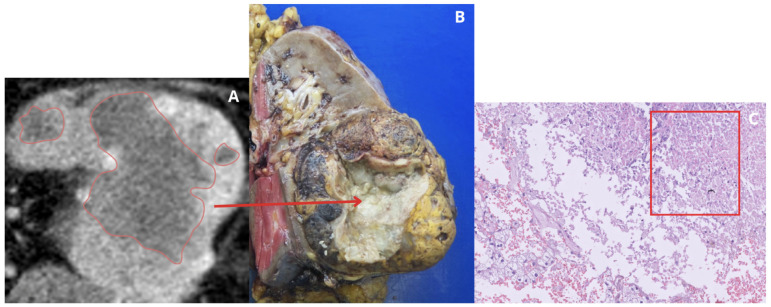
Examples of tumor necrosis in RCC. (**A**) CT image showing central hypoattenuating necrotic areas delimited by red outlines. Red arrow pointing from the main necrotic area in CT to its corresponding macroscopic correlation in the gross pathology specimen (**B**). (**C**) Microscopic appearance of coagulative necrosis (red box).

Tumor necrosis (Figure 2) is another well-established adverse prognostic factor, particularly in ccRCC [21]. The presence and extent of necrosis reflect rapid tumor growth and hypoxia-driven ischemic changes and provide prognostic information beyond conventional staging and grading parameters [22].

### 3.3. ISUP Grading System and Prognostic Significance

The International Society of Urological Pathology (ISUP) grading system, adopted by the World Health Organization, provides a standardized framework for grading RCC, specifically for clear cell and papillary subtypes. This system is based on nucleolar prominence for grades 1–3 and extreme nuclear atypia or dedifferentiation for grade 4.

**Figure 3:**
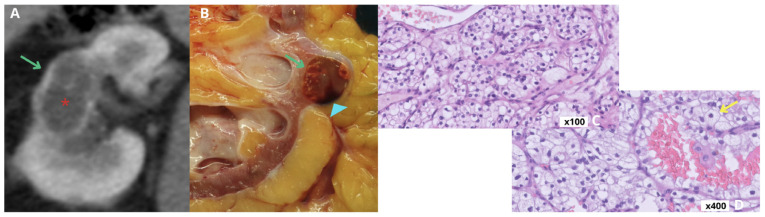
ISUP grade 1 RCC. (**A**) CT image showing a well-circumscribed renal mass (green arrow) with central hypoattenuation/cystic changes (*). (**B**) Gross pathology specimen confirming well-circumscribed tumor (green arrow) and a hemorrhagic fluid level (blue arrowhead). (**C**) Microscopic appearance at low magnification (×100) showing nests of clear cells. (**D**) High-magnification microscopy (×400) demonstrating clear cells with abundant cytoplasm and small, uniform nuclei (yellow arrow).

**Figure 4:**
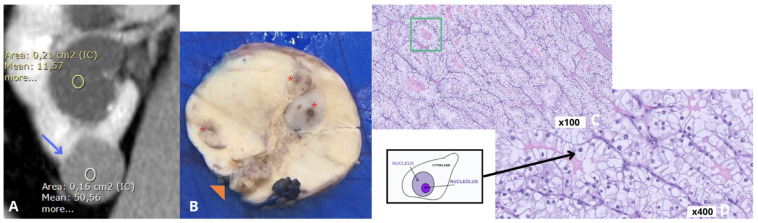
ISUP grade 2 RCC. (**A**) CT image showing an solid renal mass with mild heterogeneity (blue arrow; white ROI, 50 HU) and a renal cyst (yellow ROI, 11 HU). (**B**) Gross pathology specimen showing a solid renal mass with partially-defined borders (orange arrowhead) and focal hemorrhagic areas (*). (**C**) Microscopic view at low magnification (×100) outlining acinar architecture (green box). (**D**) High-magnification microscopy (×400) showing clear cells with visible nucleoli, linked to a representative schematic diagram (black box).

**Figure 5:**
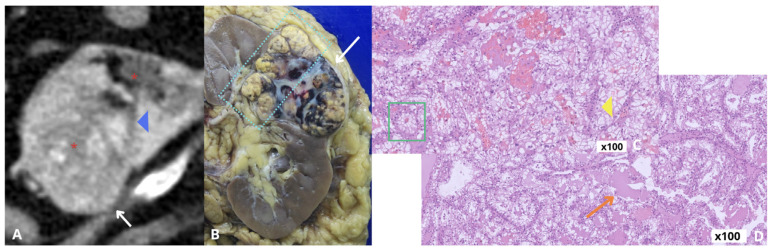
ISUP grade 3 RCC. (**A**) CT image showing a heterogeneous renal mass (white arrow) with central necrosis (*), and infiltrative features (blue arrowhead). (**B**) Gross pathology specimen confirming heterogeneous appearance of the mass with infiltrative extension into the perinephric fat and renal sinus (dashed cyan box). (**C**) Microscopic view at low magnification (×100) highlighting acinar pattern (green box) and tumor pleomorphism with prominent cellular atypia (yellow arrowhead). (**D**) Additional microscopic view (×100) demonstrating areas of focal necrosis (orange arrow) alongside high-grade tumor cells.

**Figure 6:**
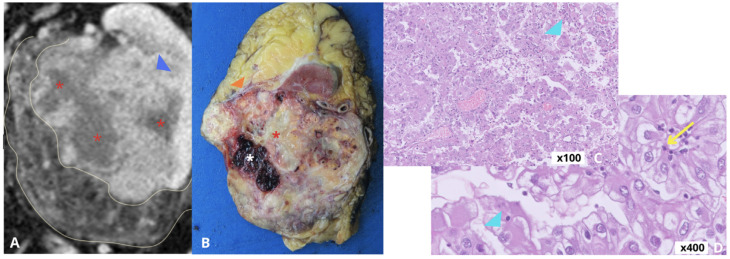
ISUP grade 4 RCC. (**A**) CT image showing a large, heterogeneous renal mass with infiltrative margins extending into the renal cortex (blue arrowhead) and perinephric fat (yellowish thin outlines), and multiple areas of necrosis (*). (**B**) Gross pathology specimen showing infiltrative extension into surrounding tissues (orange arrowhead), intratumoral hemorrhage (white *), and prominent central necrosis (red *). (**C**) Microscopic view at low magnification (×100) demonstrating marked cellular pleomorphism and rhabdoid differentiation (cyan arrowhead). (**D**) High-magnification microscopy (×400) showing rhabdoid differentiation (cyan arrowhead) and prominent nucleoli (yellow arrow).

ISUP grade 1 (Figure 3) tumors exhibit inconspicuous nucleoli visible at ×400 magnification, whereas grade 2 (Figure 4) tumors demonstrate visible and eosinophilic nucleoli at the same magnification. Grade 3 (Figure 5) tumors have prominent nucleoli identifiable at ×100 magnification. Grade 4 (Figure 6) encompasses tumors with marked nuclear pleomorphism, sarcomatoid differentiation, or rhabdoid features, which supersede nucleolar grading and associate with poor outcomes. The presence of sarcomatoid or rhabdoid differentiation mandates classification as ISUP grade 4, regardless of histologic subtype [23].

ISUP grading system is not applied to chromophobe RCC, as it does not reliably demonstrate prognostic value in this subtype [20]. The ISUP grade remains a robust and reproducible prognostic marker for clear cell and papillary RCC and is recommended for routine pathological assessments.

### 3.4. Clinical Implications on Histologic Grade and Aggressive Features

The integration of ISUP grade with histopathologic features like tumor necrosis and sarcomatoid or rhabdoid differentiation provides a framework for prognostication and clinical decision-making in RCC. A high ISUP grade (grades 3–4), extensive necrosis, and dedifferentiated components identify patients at increased risk for recurrence and cancer-specific mortality, even in localized tumors at diagnosis. These features influence postoperative surveillance intensity and support consideration of adjuvant systemic therapy in selected high-risk patients [1,24].

In advanced and metastatic RCC, histologic features carry therapeutic implications. Sarcomatoid differentiation, despite poor prognosis, has shown improved response to immune checkpoint inhibitor-based regimens, now the preferred first-line options. Current clinical guidelines advocate for intensive follow-up and tailored systemic therapy for patients with high-grade or aggressive tumors [19].

Histologic subtype, ISUP grade, tumor necrosis, and dedifferentiation patterns are key determinants of RCC biological behavior. However, these factors are often established only after tissue sampling or surgical resection, highlighting the need for noninvasive imaging biomarkers that may improve preoperative risk stratification when interpreted together with clinical and pathologic information.

## 4. Computed Tomography in the Evaluation of Renal Cell Carcinoma

### 4.1. Overview and Technical Role on CT in RCC

Contrast-enhanced multiphasic CT remains the primary imaging modality for the evaluation of renal masses and plays a central role in the diagnosis, staging, and surgical planning of RCC, with reported sensitivity of 88% and specificity of 75% [3,25]. Standard renal mass protocols typically include unenhanced, corticomedullary, nephrographic, and excretory phases, enabling assessment of lesion enhancement, internal architecture, calcifications, hemorrhage, local extension, vascular invasion, and metastatic disease [26].

Triple-phase CT of the chest, abdomen, and pelvis is the standard imaging strategy for RCC staging, with accuracy of up to 91% for local staging [17]. CT is also essential for treatment planning, providing detailed information on tumor size, location, collecting system involvement, renal sinus invasion, and vascular anatomy, particularly when nephron-sparing surgery is being considered [17,27].

Despite its widespread use and excellent diagnostic performance, conventional CT has important limitations for lesion characterization. Significant imaging overlap exists among RCC subtypes and benign lesions such as oncocytoma and lipid-poor angiomyolipoma (Table 2). Furthermore, CT cannot reliably predict histologic subtype, tumor grade, or biological aggressiveness in all cases. These limitations support a complementary diagnostic approach that may include quantitative imaging biomarkers, advanced imaging techniques, and renal mass biopsy when results are expected to influence management [8,9,17].

### 4.2. Morphological CT Features Associated with Non-Aggressive Renal Tumors

Less aggressive renal tumors frequently demonstrate well-defined margins, relatively homogeneous internal architecture, and gradual enhancement patterns on multiphasic CT. These characteristics are commonly observed in papillary RCC, chromophobe RCC, and several benign renal lesions.

**Figure 7:**
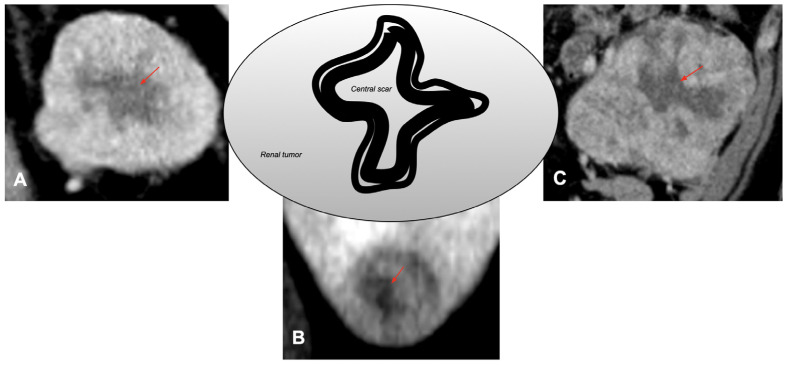
Central scar appearance on CT (red arrows) in oncocytoma (**A**), chromophobe RCC (**B**), and clear cell RCC (**C**), illustrating the limited specificity of this finding.

Papillary RCC typically appears as a hypovascular mass with low-level enhancement relative to the adjacent renal cortex. Chromophobe RCC and oncocytoma often demonstrate moderate and relatively homogeneous enhancement. Although a central stellate scar may be observed in oncocytoma (Figure 7), this finding lacks sufficient specificity to reliably distinguish benign from malignant lesions [28,29,30].

The absence of necrosis, vascular invasion, infiltrative margins, and regional lymphadenopathy generally supports a less aggressive imaging phenotype. However, substantial overlap exists between indolent malignancies and benign tumors, limiting the specificity of these findings [8,28,29,31].

### 4.3. Morphological CT Features Associated with Aggressive Renal Tumors

**Figure 8:**
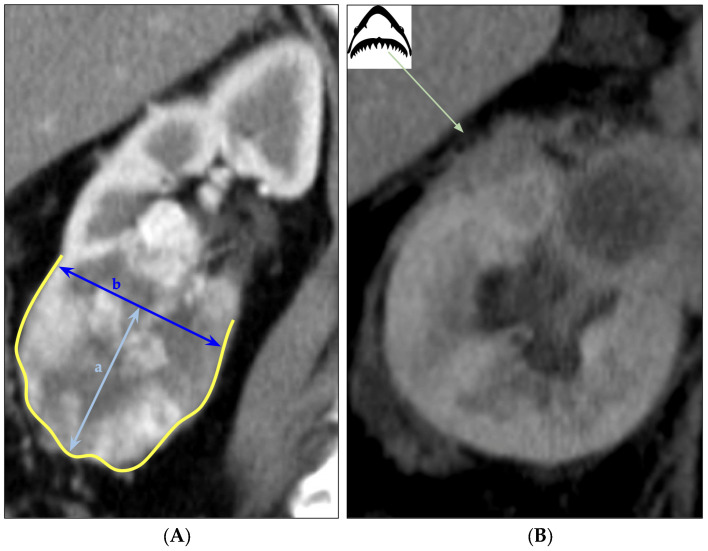
Deep lobulation and saw-tooth contour on CT. (**A**) Deeply lobulated tumor contour outlined in yellow; the arc-chord ratio can quantify this lobulation, where the light blue double-headed arrow (a) represents the arc depth and the dark blue double-headed arrow (b) represents the chord length. (**B**) Sharp acute projections at the tumor–parenchyma interface representing the saw-tooth sign, highlighted by a light green arrow connecting the findings to an explanatory schematic diagram of a shark’s jaw morphology.

Although no individual CT feature is pathognomonic of aggressive RCC, several findings correlate with adverse pathological characteristics. These include large tumor size, infiltrative margins, marked heterogeneity, tumor necrosis, irregular enhancement, intralesional calcifications, collecting system involvement, regional lymphadenopathy, and signs of renal capsule invasion such as deep lobulation or the “saw-tooth” sign (Figure 8) [3,29,31,32,33,34].

**Figure 9:**
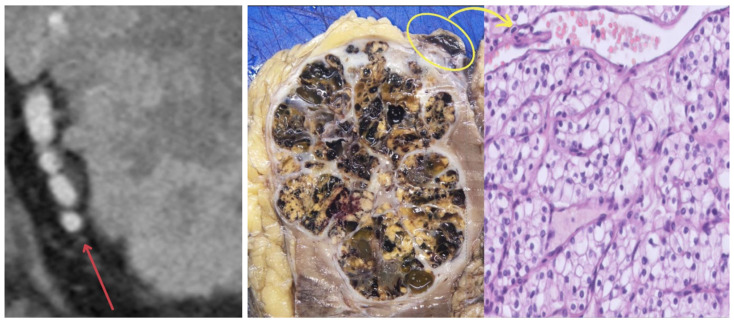
Prominent peritumoral vessels around RCC (red arrow), an imaging marker associated with angiogenesis and aggressive behavior.

Peritumoral neovascularity (Figure 9) has emerged as a particularly relevant imaging biomarker. Enlarged vessels surrounding the tumor reflect increased angiogenic activity, and correlate with higher ISUP grade, advanced pathological stage, and worse oncologic outcomes, even at early stages [34,35,36,37,38,39].

**Figure 10:**
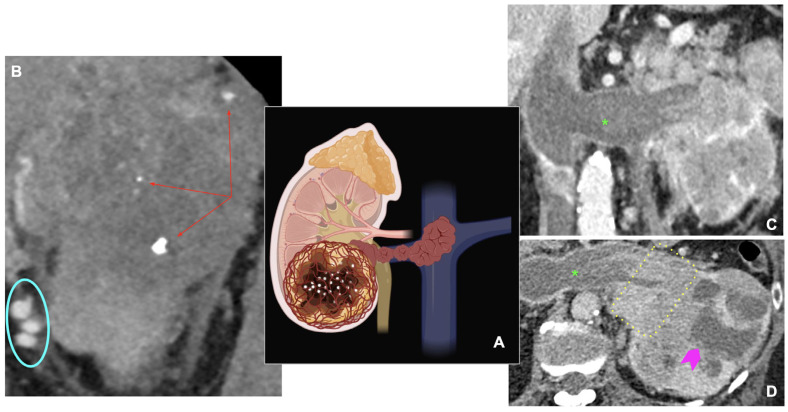
CT findings associated with aggressive RCC. (**A**) Schematic diagram illustrating key features of tumor aggressiveness (Image created with BioRender.com). (**B**) CT scan showing multiple focus of intratumoral calcifications (red arrows) and prominent, tortuous peritumoral vessels (cyan oval). (**C**) Coronal CT reconstruction showing a venous tumor thrombus (green *) extending through the renal vein. (**D**) Axial CT image demonstrating extensive renal sinus infiltration (dashed yellow box), a venous tumor thrombus (green *), and a large area of internal hypodense tumor necrosis (purple arrowhead).

CT is also highly effective for detecting venous invasion and extension into the renal vein or inferior vena cava (Figure 10), findings that significantly influence staging, prognosis, and surgical management. The combination of venous invasion and perinephric fat infiltration is associated with particularly unfavorable outcomes [40,41].

### 4.4. Dynamic CT Features

Quantitative parameters derived from multiphasic CT have been investigated as imaging biomarkers of tumor aggressiveness. Commonly studied metrics include mean unenhanced attenuation (HU), enhancement-related measurements such as attenuation difference (Delta HU), tumor-to-cortex ratio (TCR), difference attenuation ratio (DAR), and volumetric heterogeneity metrics.

**Figure 11:**
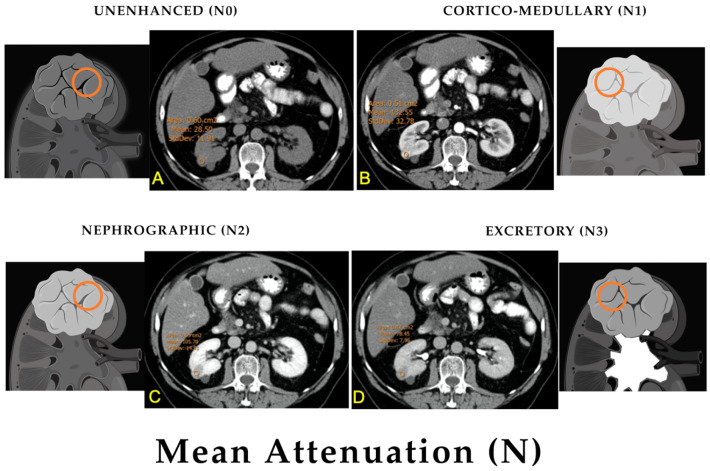
Mean attenuation (N) measured across multiphase CT. Schematic kidney diagrams accompany each phase, where the orange circles highlight a Region of interest (ROI) of the renal tumor (images created with BioRender.com). (**A**) Unenhanced phase (N0) showing the baseline attenuation of the lesion. (**B**) Corticomedullary phase (N1) demonstrating early contrast enhancement. (**C**) Nephrographic phase (N2) displaying peak or plateau parenchymal enhancement. (**D**) Excretory phase (N3) showing progressive contrast washout. Images created with BioRender.com.

Mean attenuation reflects tumor density on unenhanced CT (Figure 11). Aggressive RCCs, particularly high-grade ccRCC, show higher unenhanced attenuation values, due to increased cellularity and reduced microscopic fat content. Benign and indolent tumors often demonstrate lower baseline attenuation [42].

**Figure 12:**
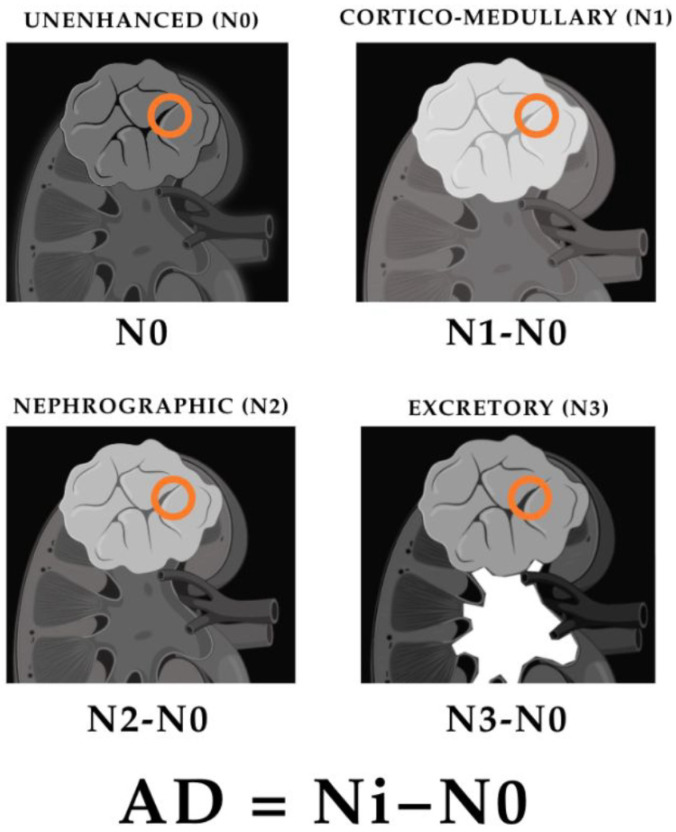
Schematic kidney diagrams accompany each phase, where the orange circles highlight a Region of interest (ROI) of the renal tumor (images created with BioRender.com). Attenuation difference (AD), calculated as the difference between the attenuation of each contrast-enhanced phase and unenhanced attenuation (AD = Ni − N0).

Attenuation difference (ΔHU) serves as a surrogate for tumor vascularity and enhancement (Figure 12). Higher values are associated with less aggressive biology and improved disease-free survival, whereas lower enhancement correlates with higher grade and increased recurrence risk [43].

**Figure 13:**
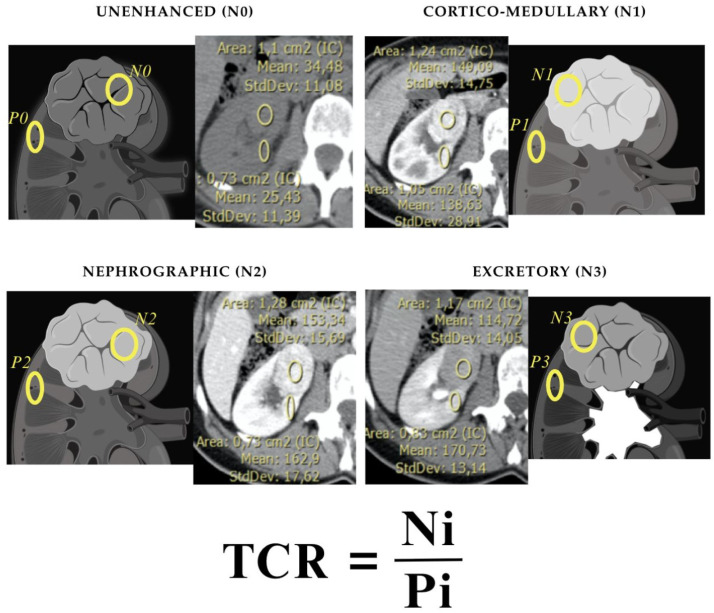
Tumor-to-cortex ratio (TCR), calculated as the ratio between lesion attenuation in each CT phase (Ni) and normal cortical attenuation in the same phase (Pi). Images created with BioRender.com.

**Figure 14:**
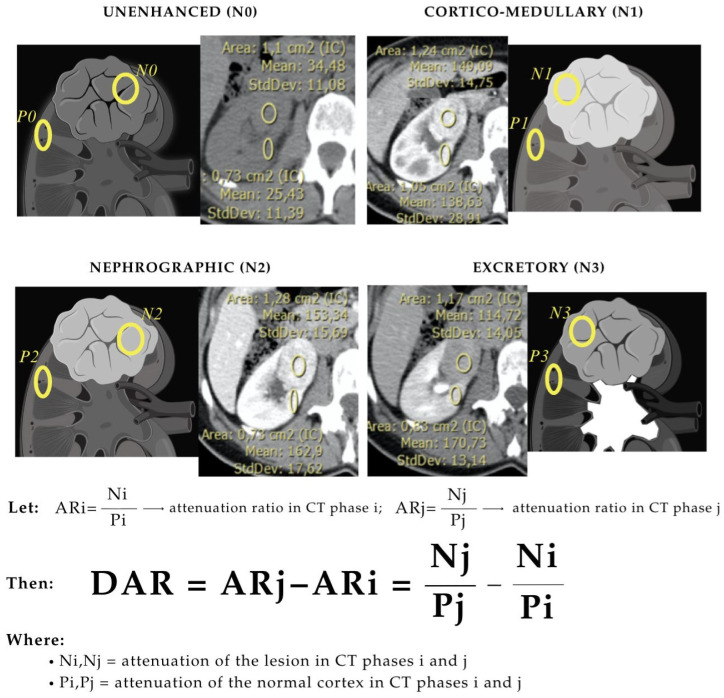
Difference attenuation ratio (DAR), calculated as the difference between attenuation ratios across CT phases: DAR = ARj − ARi = Nj/Pj − Ni/Pi, where Ni and Nj indicate lesion attenuation and Pi and Pj indicate normal cortical attenuation in CT phases i and j. Images created with BioRender.com.

TCR (Figure 13) and DAR (Figure 14) normalize tumor enhancement to the adjacent renal parenchyma. Lower ratios and differences characterize aggressive RCC, while benign and indolent tumors exhibit higher ratios, reflecting more avid enhancement and preserved vascularity [42,44,45].

Volumetric histogram analysis refines this assessment. Increased entropy and standard deviation within tumor volume indicate greater heterogeneity, a hallmark of aggressive ccRCC, whereas benign lesions display more homogeneous enhancement profiles [46].

Although these parameters are clinically attractive, reported thresholds vary considerably among studies. Their routine use remains limited by differences in CT acquisition, reconstruction, contrast timing, segmentation, and validation strategy.

### 4.5. Role of Renal Mass Biopsy

Renal mass biopsy has emerged as a valuable adjunct to imaging in the characterization of indeterminate renal masses [8]. This is particularly relevant because a substantial proportion of solid enhancing renal masses are benign or indolent, and histologic confirmation can help avoid overtreatment and support individualized management strategies [17].

Contemporary percutaneous renal mass biopsy demonstrates high diagnostic performance for differentiating benign from malignant lesions, with reported sensitivity of 98–99% and specificity of 94–100% [17]. Important limitations include nondiagnostic sampling, intratumoral heterogeneity, and lower accuracy for assigning grade compared with surgical pathology.

Current practice therefore emphasizes the complementary roles of imaging and biopsy. CT provides anatomical characterization, staging information, and assessment of tumor extent, whereas biopsy offers histologic confirmation and additional risk stratification. The American Urological Association recommends renal mass biopsy when results are likely to influence management, particularly before active surveillance, ablative therapies, or systemic treatment selection [6,17].

### 4.6. Summary

CT remains the cornerstone of RCC evaluation, providing essential information for diagnosis, staging, and treatment planning. Morphologic and quantitative imaging features can suggest tumor subtype and biological aggressiveness, but overlap among renal tumor entities limits definitive characterization. Contemporary assessment therefore benefits from an integrated approach in which CT, MRI or CEUS when appropriate, renal mass biopsy, and multidisciplinary review are used according to the clinical question. These challenges have stimulated interest in radiomics and other quantitative methods designed to extract additional biologically relevant information from routine imaging.

## 5. CT and ISUP Grade: Radiological and Pathological Correlation

Accurate preoperative assessment of tumor grade remains a critical challenge in the management of RCC. Several studies have evaluated the diagnostic performance of CT imaging features alone, excluding radiomics, for predicting ISUP grade, reporting moderate accuracy with area under the curve (AUC) values from 0.75 to 0.85, sensitivity between 58% and 71%, and specificity from 79% to 90% [47,48]. These figures underscore the potential utility of CT-based assessment, while highlighting its limitations compared to histology.

### 5.1. Diagnostic Performance and Key Imaging Features

CT features most associated with ISUP grade include tumor size, attenuation values, enhancement patterns, and normalized iodine concentration (particularly in dual-energy CT). A multivariable model incorporating transverse diameter, unenhanced attenuation, and normalized iodine concentration achieved an AUC of 0.85, sensitivity of 70%, specificity of 90%, and overall accuracy of 82% for distinguishing low-grade from high-grade ccRCC [49]. Interobserver agreement for CT-based algorithms is generally good, with weighted kappa values around 0.71 for features such as mass-to-cortex attenuation ratio and heterogeneity score. Performance declines in small renal masses and in certain CT phases, such as the nephrographic phase, where feature discrimination is less pronounced [48].

### 5.2. Limitations and the Need for Quantitative Imaging

Despite its value, CT-based grading remains limited by moderate sensitivity, interobserver variability, and qualitative visual assessment. These limitations underscore the need for advanced imaging approaches capable of extracting deeper biologic information from routinely acquired CT datasets.

Radiomics shifts from qualitative interpretation to high-dimensional quantitative analysis. Several studies have reported improved performance when radiomic features are combined with clinical and imaging variables, achieving AUC values exceeding 0.90 for ISUP grade prediction [47,50]. These findings suggest that radiomics may provide complementary information beyond conventional visual assessment. However, most available evidence derives from retrospective investigations with considerable methodological heterogeneity, emphasizing the need for external validation before clinical implementation.

## 6. Radiomics in Renal Cell Carcinoma

### 6.1. Radiomics Concept and Workflow

Radiomics refers to the high-throughput extraction of quantitative features from medical images to convert imaging into mineable data for precision oncology [51,52]. Unlike visual interpretation, which is qualitative and observer-dependent, radiomics enables objective quantification of tumor phenotypes by analyzing patterns of intensity, shape, texture, and heterogeneity within a region (ROI) or volume (VOI) of interest [53].

**Figure 15:**
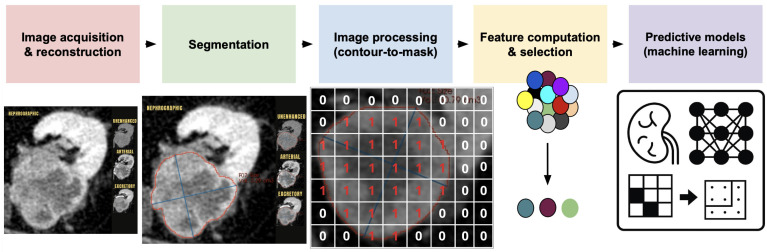
Radiomics workflow from image acquisition and segmentation to feature extraction, model development, and validation.

A standard radiomics pipeline includes the following (Figure 15): (1) standardized image acquisition; (2) tumor segmentation; (3) extraction of handcrafted features, including first-order, shape-based, and texture-based metrics; (4) feature selection to remove redundancy; and (5) development and validation of predictive models [52,53,54,55].

Radiomic features fall into four categories: shape-based features, first-order features, second-order (texture) features, and higher-order features from filtered images [56,57] (Table 3). Features are selected based on reproducibility and relevance for building predictive models for diagnosis, prognosis, and treatment response. Dimensionality reduction and cross-validation help avoid overfitting and ensure generalizability [58].

These features are extracted from ROIs or VOIs and interpreted in relation to tumor biology, heterogeneity, and morphology. Standardization efforts such as the Image Biomarker Standardization Initiative (IBSI) provide reproducible feature definitions and should be used when developing or comparing radiomics models [59].

Radiomics has shown potential across several oncology settings, including lung, breast, prostate, hepatobiliary, and abdominopelvic malignancies. In selected research cohorts, radiomic signatures may improve diagnostic accuracy, risk stratification, and treatment-response prediction when combined with clinical and imaging variables [60,61,62].

### 6.2. Radiomics for Histologic Subtype Classification in RCC

One of the earliest clinical applications of radiomics in renal imaging was the differentiation of RCC subtypes and benign renal masses. Several studies suggest that handcrafted radiomic features from contrast-enhanced CT may help differentiate clear cell, papillary, and chromophobe RCC, as well as RCC from oncocytoma, although reported performance varies substantially (Table 4) [63,64,65,66].

Texture-based features, including entropy, contrast and heterogeneity metrics, emerge as key discriminators, reflecting differences in tumor microarchitecture and heterogeneity [64,65,67,68,69]. CcRCC exhibits greater textural variability than papillary RCC, which often shows lower enhancement and more homogeneous architecture [63,64]. For benign lesions, although oncocytoma remains difficult to characterize, lipid-poor angiomyolipoma is often recognized with higher accuracy [63].

Combined radiomics models integrating texture, shape, and intensity features have reported encouraging but heterogeneous performance for differentiating renal tumor subtypes, with AUC values ranging from approximately 0.80 in multicenter studies to above 0.90 in selected single-center binary analyses [65,69,70]. Performance is influenced by cohort composition, imaging protocol, tumor segmentation, model design, and validation strategy. External validation remains limited, restricting generalizability.

In clinical practice, accurate subtype prediction using imaging alone remains challenging. CT-based radiomics may provide complementary information, particularly for small or indeterminate renal masses, but should not replace established imaging assessment or histopathologic confirmation when biopsy is clinically indicated. MRI-based radiomics and multimodal AI approaches may add functional and clinical information, but they require harmonized protocols and external validation similar to CT-based models.

### 6.3. Radiomics for Predicting ISUP Grade and Tumor Aggressiveness

Recent meta-analyses have reported promising performance for radiomics-based models in predicting tumor grade and aggressive histopathologic features (Table 5), with pooled AUC values generally ranging between 0.84 and 0.88 [71,72]. Combined models integrating radiomics with clinical variables may improve performance, with pooled AUCs up to 0.90 and higher sensitivity for detecting high-grade disease [72]. However, these estimates should be interpreted cautiously because included studies often differ in sample size, CT phase, segmentation method, feature extraction, and validation design.

Radiomics features associated with intratumoral heterogeneity have been linked to aggressive histopathologic characteristics in RCC. High entropy, increased variance, and low homogeneity on CT or MRI may reflect necrosis, high-grade histology, and disordered tissue organization [34]. Sarcomatoid differentiation has also been associated with increased textural complexity, including elevated GLCM contrast, cluster shade, run-length non-uniformity, and irregular shape features [73,74]. These associations are biologically plausible but remain insufficiently standardized for direct clinical decision-making.

Microvascular invasion has been investigated using composite radiomics signatures that integrate texture-based metrics with first-order intensity features. These models may capture imaging correlates of vascular infiltration that are not visible on conventional imaging, but clinical use requires external validation and transparent reporting [75,76].

In summary, radiomics-based models may complement conventional imaging for assessing tumor aggressiveness in renal tumors, especially when combined with clinical data. At present, they should be regarded as investigational decision-support tools rather than ready replacements for established clinical, imaging, and histopathologic assessment.

### 6.4. Prognostic Radiomics: Recurrence, Survival and Treatment Response

Radiomics-based tumor heterogeneity scores derived from texture features have been associated with recurrence and poor clinical outcome in retrospective cohorts [76,77,78]. Texture features such as entropy, cluster shade, and GLCM metrics quantify tumor heterogeneity, associated with aggressive biology and recurrence risk [76,78]. Shape features and intensity features have been independently linked to survival [79,80]. Wavelet-transformed texture features enhance prognostic accuracy by multi-scale heterogeneity [81].

Growing evidence suggests that radiomics signatures may provide incremental prognostic information for overall survival and recurrence risk beyond established clinical models such as TNM, SSIGN, and Leibovich scores [71,76,78]. However, most evidence remains retrospective, and prospective validation is required before routine clinical use can be recommended. Decision curve analyses suggest that radiomics-based models may offer clinical benefit for adjuvant therapy or surveillance decisions in selected high-risk populations when combined with clinical data, but these findings still require prospective testing [76].

RCC treatment faces major challenges, including resistance to tyrosine kinase inhibitors and variable responses to immunotherapy, which are influenced by tumor microenvironment dynamics and immune infiltration patterns [82,83]. Preliminary studies suggest that radiomic features may help predict response to immune checkpoint inhibitors and characterize the tumor immune microenvironment. Higher tumor intensity variance, maximum intensity, and non-uniformity on baseline CT have been associated with progression and poor immunotherapy response [84]. Other features, including energy, run-length non-uniformity, busyness, and gray-level non-uniformity, have been investigated for identifying minimal residual disease after immune checkpoint blockade [85]. Radiomics-derived heterogeneity has also been correlated with immune phenotypes, including tumor-infiltrating CD8+ cells and response to anti-PD-1/PD-L1 therapy [86]. These observations remain preliminary and require validation, standardization, and prospective clinical testing before implementation [87,88].

### 6.5. Limitations and Challenges of Radiomics in RCC

Radiomics in RCC remains limited by methodological heterogeneity. Differences in CT acquisition and reconstruction, contrast timing, segmentation strategies, feature extraction, and machine-learning workflows reduce reproducibility and generalizability across institutions. Most studies are retrospective and single-center, frequently without standardized imaging protocols or external validation, resulting in inconsistent performance and limited real-world applicability [88].

Model robustness is further affected by variable methodological quality and small datasets. Low Radiomics Quality Scores often reflect limited prospective validation, calibration, decision-curve analysis, open-source code, data sharing, phantom or test–retest repeatability assessments, and transparent reporting. Manual and semi-automated segmentation introduce feature instability, while high-dimensional feature sets increase the risk of overfitting; internal cross-validation alone is insufficient without external testing across scanners, vendors, reconstruction protocols, and institutions [69,88,89,90].

Clinical translation therefore requires interpretable, auditable, and workflow-compatible models that demonstrate added value beyond established clinical, imaging, and histopathologic pathways. Future studies should use harmonized acquisition and segmentation pipelines, IBSI-compliant feature definitions, open-science practices, and prospective multicenter validation, with reporting aligned with CLAIM 2024 and TRIPOD+AI [50,88].

## 7. Future Steps for Characterization of Renal Cell Carcinoma and Clinical Usage

Future progress in RCC imaging will depend on improved standardization, reproducibility, and prospective validation of quantitative imaging biomarkers. Harmonized acquisition protocols, robust segmentation methods, IBSI-compliant feature definitions, and adherence to reporting standards such as CLAIM 2024 and TRIPOD+AI are essential for improving the reliability of radiomics and AI research.

Emerging technologies, including photon-counting CT, molecular imaging, and multimodal AI models integrating imaging, clinical, pathological, and molecular data, may further enhance noninvasive RCC characterization [9,91]. Photon-counting CT may improve spatial resolution and spectral information, while molecular imaging may help interrogate tumor biology beyond morphology. However, these methods should currently be viewed as complementary and investigational until validated clinical pathways are established.

A pragmatic clinical pathway is therefore stepwise: multiphasic CT for detection, staging, and surgical planning; MRI, CEUS, or DECT for selected indeterminate lesions or functional characterization; renal mass biopsy when histology is expected to change management; and radiomics, molecular imaging, photon-counting CT, or multimodal AI as research-supported decision aids within multidisciplinary frameworks until prospective validation and regulatory integration support routine use.

## 8. Conclusions

Multiphasic CT remains the cornerstone of RCC characterization, staging, and surgical planning. Conventional imaging provides valuable information regarding tumor subtype and aggressiveness, although important limitations remain in predicting tumor biology before treatment.

Radiomics and other quantitative imaging approaches may capture imaging biomarkers associated with histologic subtype, tumor grade, and biological aggressiveness. While numerous studies have reported encouraging results, current evidence is predominantly retrospective and heterogeneous, and important challenges related to reproducibility, segmentation, standardization, external validation, and interpretability remain unresolved.

Future advances will depend on multicenter collaboration, harmonized imaging protocols, adherence to methodological and reporting standards, and prospective validation studies. Integration of quantitative imaging with clinical, pathological, and molecular information may ultimately support more personalized management of patients with RCC, provided that these tools demonstrate added value in real clinical workflows.

## Figures and Tables

**Table 1:** Principal imaging tools for renal tumor assessment.

	US/CEUS	CT/DECT	MRI
Protocol	B-mode, Doppler; CEUS with microbubble contrast	Multiphase CT (non-contrast, corticomedullary, nephrographic, excretory); DECT with iodine quantification and virtual unenhanced images (VUE)	T1/T2-weighted, DWI, dynamic contrast-enhanced, subtraction, delayed post-contrast
Clinical applications	Initial detection, cyst vs. other lesions, follow-up, CEUS for vascularity and enhancement	Characterization, staging, surgical planning, DECT for enhancement and cyst vs. solid	Indeterminate lesions, venous involvement, subtyping, cystic lesion characterization, follow-up
Advantages	Widely available, no radiation, safe in renal impairment	High sensitivity/specificity, staging, DECT reduces extra imaging and radiation	Superior soft tissue contrast, functional imaging, no radiation, better for venous involvement
Disadvantages	Operator-dependent, limited for complex/indeterminate masses	Radiation, contrast nephrotoxicity, limited for benign vs. malignant, DECT VUE may underestimate attenuation	Gadolinium risk in severe renal dysfunction, less sensitive for calcifications, costly

US—ultrasound; CEUS—contrast-enhanced ultrasound; CT—computed tomography; MRI—magnetic resonance imaging; DWI—diffusion-weighted imaging; DECT—dual-energy computed tomography; VUE—virtual unenhanced images.

**Table 2:** CT imaging features associated with non-aggressive and aggressive renal tumors.

CT Imaging Feature	Lower-Risk Pattern	Higher-Risk Pattern	Clinical/Pathologic Implication
Size	Small to moderate	Often large or rapidly growing	Imperfect surrogate of biology but relevant for management
Margins and contour	Well circumscribed; smooth or mildly lobulated	Ill-defined, infiltrative, deeply lobulated, or saw-tooth contour	Suggests capsular, sinus, or perinephric invasion
Internal architecture	Relatively homogeneous	Marked heterogeneity, hemorrhage, or necrosis	Reflects dedifferentiation and adverse biology
Enhancement pattern	Gradual or preserved enhancement	Irregular, heterogeneous, or reduced enhancement	Associated with grade/recurrence in selected studies; overlap is common
Peritumoral neovascularity	Absent or minimal	Enlarged or multiple peritumoral vessels	Associated with higher grade, advanced pT stage, and worse outcome
Quantitative attenuation/enhancement	Lower unenhanced HU; higher Delta HU, TCR, or DAR	Higher unenhanced HU; lower Delta HU, TCR, or DAR	May reflect cellularity, microscopic fat, vascularity, and tumor-cortex contrast
Necrosis and calcification	Absent or limited	Necrosis and/or calcifications present	Necrosis is an adverse prognostic factor; calcification may indicate advanced disease
Local extension	No venous, sinus, collecting-system, or perinephric invasion	Renal vein/IVC thrombus, renal sinus or perinephric invasion, collecting-system involvement	Defines advanced stage and surgical complexity
Regional lymph nodes	Absent	Suspicious lymphadenopathy	Supports advanced disease but requires clinical correlation

ccRCC—clear cell Renal Cell Carcinoma; DAR—Difference Attenuation Ratio; HU—Hounsfield Units; ISUP—International Society of Urological Pathology; OS—Overall Survival; PTN—Peritumoral Neovascularity; pT—pathological Tumor staging; TCR—Tumor-to-cortex ratio.

**Table 3:** Common radiomics features: classification, definition, and clinical interpretation.

Feature Category	Examples	Definition	Clinical Interpretation
First-order intensity	Mean, entropy, skewness, kurtosis	Distribution of voxel values within the ROI/VOI	May reflect density, necrosis, and global heterogeneity
Shape-based	Volume, surface area, sphericity	Three-dimensional tumor size and geometry	Irregularity may suggest infiltrative growth
GLCM texture	Contrast, homogeneity, correlation	Spatial relationship of neighboring gray levels	Captures heterogeneity and tissue organization
GLRLM/GLSZM texture	Short-run emphasis, non-uniformity, zone-size variance	Patterns of repeated gray levels or homogeneous zones	May reflect fine/coarse texture and architecture disorder
Higher-order/filter-based	Wavelet energy	Features after image filtering or frequency decomposition	May capture subtle multi-scale patterns; interpretation is less direct

GLCM—gray-level co-occurrence matrix; GLRLM—gray-level run-length matrix; ROI—Region of Interest.

**Table 4:** Radiomics Findings by Histologic Subtype in Renal Tumors (CT-Based Radiomics).

Histologic Subtype	Radiomics Pattern Commonly Reported	Performance Summary	Main Limitations
ccRCC	Greater heterogeneity; texture, shape, and wavelet-derived features	AUC around 0.75 in multiclass external validation; higher in selected binary or single-center studies	Performance decreases in multiclass settings and depends on acquisition/reconstruction
pRCC	Hypovascular and relatively homogeneous texture/intensity features	Often good binary performance, lower multiclass accuracy	Overlap with oncocytoma and chromophobe RCC
chRCC/oncocytoma	Subtle texture and shape differences; central-scar matched models reported	High AUC in dedicated binary tasks, but variable across cohorts	Limited generalizability and substantial imaging overlap
RO and LP-AML	Low-grade heterogeneity or distinct fat-poor AML intensity/texture signatures	RO remains challenging; LP-AML often performs better in selected models	Requires careful validation against benign mimics
Overall	Radiomics may add information to clinical and imaging variables	Best results occur in focused tasks with robust validation	External validation, standardization, and clinical impact remain limited

Reported AUC ranges depend on study design, cohort size, imaging phase, and presence of external validation. AUC—Area Under the Curve; ccRCC—clear cell Renal Cell Carcinoma; chRCC—chromophobe Renal Cell Carcinoma; LP-AML—Lipid-Poor Angiomyolipoma; pRCC—papillary Renal Cell Carcinoma; RO—Renal Oncocytoma.

**Table 5:** Radiomics Features Correlated with Aggressive Histological Factors in RCC.

Feature Group	Examples	Associated Aggressive Factor(s)	Interpretation and Limitations
First-order intensity	Median intensity, variance, skewness, kurtosis	Necrosis, high grade	Summarizes intensity distribution; sensitive to acquisition and contrast timing
GLCM texture	Entropy, contrast, homogeneity, cluster shade	Necrosis, sarcomatoid differentiation, high grade	Reflects heterogeneity and tissue disorganization; requires IBSI-compliant definitions
GLRLM/GLSZM texture	Run-length non-uniformity, gray-level non-uniformity, zone-size variance	Sarcomatoid differentiation, microvascular invasion	Captures repeated gray-level patterns and architectural disorder
Shape features	Sphericity, flatness, area density	Invasion, high grade, poor prognosis	May reflect infiltrative or irregular growth; segmentation dependent
Higher-order features	Wavelet energy and filtered features	High grade, necrosis, recurrence	May capture multi-scale heterogeneity but is less intuitive clinically
Composite signatures	Selected radiomics plus clinical variables	Microvascular invasion, recurrence, survival	Potentially useful decision support; requires external validation and calibration

GLCM—gray-level co-occurrence matrix; GLRLM—gray-level run-length matrix; GLSZM—gray-level size zone matrix.

## Data Availability

No new data were generated or analyzed for this review. All information discussed is included in the manuscript and in the cited literature.
